# Response to “Comment on ‘Individual and Neighborhood Socioeconomic Status and the Association between Air Pollution and Cardiovascular Disease’”

**DOI:** 10.1289/EHP1268

**Published:** 2017-01-01

**Authors:** Gloria C. Chi, Anjum Hajat, Kristin A. Miller, Eric A. Whitsel, Beth Ann Griffin, Regina A. Shih, Joel D. Kaufman

**Affiliations:** 1 Department of Epidemiology, School of Public Health, University of Washington, Seattle, Washington, USA; 2 Department of Epidemiology, Gillings School of Global Public Health, University of North Carolina at Chapel Hill, Chapel Hill, North Carolina, USA; 3 Department of Medicine, University of North Carolina at Chapel Hill, Chapel Hill, North Carolina, USA; 4 RAND Corporation, Arlington, Virginia, USA; 5 Department of Environmental and Occupational Health Sciences, School of Public Health, University of Washington, Seattle, Washington, USA

We thank Ahmed et al. for their interest in our article. The correspondents comment on the generalizability of study results to a broader population and emphasize the need to consider racial/ethnic and sex disparities when examining the role of socioeconomic status (SES) on the relationship between air pollution and cardiovascular disease (CVD). We wish to clarify that while the Women’s Health Initiative cohort does include mostly white women, we did not exclude participants for our study on the basis of race or ethnicity. Approximately 13% of our study participants were racial/ethnic minorities, and more than half of that proportion were black. The Women’s Health Initiative is a large and important study of older women but cannot by itself inform questions of potential sex differences in susceptibility to the health effects of air pollution. Seen from a historical perspective, planning for the Women’s Health Initiative observational study cohort and related clinical trials began in the 1980s at a time when health studies had largely enrolled male participants only (Hays et al. 2003). We agree it is likely that future studies including different distributions of demographic groups might yield useful information.

The correspondents were also concerned that the enrollment of participants between 1993 and 1998 might limit the applicability of study results to current populations because of female-specific CVD guidelines released in 1999 and 2004, which were followed by a steep decline in CVD mortality among women. To clarify our design, participants entered the study from 1993 to 1998 and were followed from baseline until the end of follow-up of the main cohort in September 2005, with a mean follow-up of 7.6 years. Both guidelines were released within the range of time that participants were under study. Moreover, our study period (1993–2005) also overlaps with most of the years that were cited by Ahmed et al. as showing a sharp decrease in CVD mortality in women (1997–2009). Although CVD rates have declined over the years, CVD is still the leading cause of death and morbidity in the United States for both women and men (Heron 2016), and a reduction in CVD rates does not necessarily change the etiology of CVD as pertaining to air pollution and SES or alter the relationships between air pollution, CVD, and SES.

The correspondents attribute the drop in CVD rates in women to improvements in female CVD awareness, prevention, and treatment, and they speculate that findings might be different in a more contemporary cohort. We would like to point out that those with lower SES experience reduced access to health care and health education (Kreatsoulas and Anand 2010). Thus, women with low SES may not benefit from advancements in female CVD awareness, prevention, and treatment to the same extent as those with higher SES. It is possible that disparities in health care access and health education resulting from SES may even contribute to increased susceptibility to air pollution–related disease among persons with low SES.

Finally, the correspondents commented on the exclusion of participants with prevalent CVD at baseline. Our main objective was to understand the role of SES in confounding or modifying the relationship between air pollution and incident cardiovascular disease that has been observed in epidemiological studies. That is, our goal was to study incident disease to shed light on CVD development. Moreover, excluding those with prevalent diseases reduces potential bias related to selective survival or mortality among women with prevalent diseases. Studying prevalent CVD was not the focus of our paper, although we agree that studies of those with preexisting CVD could yield insight into susceptibility to CVD progression and important health outcomes.

## References

[r1] Hays J Hunt JR Hubbell FA Anderson GL Limacher M Allen C 2003 The Women’s Health Initiative recruitment methods and results. Ann Epidemiol 13 9 suppl S18 S77, doi:10.1016/S1047-2797(03)00042-5 14575939

[r2] Heron M 2016 Deaths: leading causes for 2014. Natl Vital Stat Rep 65 5 1 96, PMID: 27376998

[r3] Kreatsoulas C Anand SS 2010 The impact of social determinants on cardiovascular disease. Can J Cardiol 26 suppl C 8C 13C, PMID: 20847985 10.1016/s0828-282x(10)71075-8 PMC2949987

